# Glaucoma, More than Meets the Eye: Patterns of Demyelination Revealed in Human Postmortem Glaucomatous Optic Nerve

**DOI:** 10.14336/AD.2024.0336-1

**Published:** 2024-10-01

**Authors:** Gabriella E. Parrilla, Akanksha Salkar, Roshana Vander Wall, Vivek Gupta, Stuart L. Graham, Yuyi You

**Affiliations:** ^1^Department of Clinical Medicine, Faculty of Medicine, Health and Human Sciences, Macquarie University, Wallumattagal Campus, NSW 2109, Australia.; ^2^Save Sight Institute, University of Sydney, Sydney, NSW 2000, Australia.

**Keywords:** Glaucoma, Demyelination, Neurodegeneration, Optic Nerve

## Abstract

Glaucoma is a neurodegenerative disease affecting millions worldwide, characterised by retinal ganglion cell (RGC) degeneration which leads to blindness in more advanced cases. Although the pathogenesis and underlying mechanisms of glaucoma are not fully understood, there are theories that hint at demyelination playing a role in the disease process. Demyelination, or the degeneration of the myelin sheath surrounding axons, has been found in previous studies using animal models of glaucoma and clinical assessments of glaucoma patients. However, this has not been fully realised or quantified in glaucoma patients. Utilising postmortem optic nerve samples from glaucoma and healthy subjects, various immunohistochemical and morphological assessments were performed to determine the extent, if any, of demyelination in glaucomatous optic nerves. Our findings revealed that alongside nerve shrinkage and degeneration of nerve tissue fascicles, there were significantly less myelin proteins, specifically myelin basic protein (MBP), in glaucoma optic nerves. Additionally, the loss of MBP was correlated with decreased oligodendrocyte (OLG) precursors and increasing glial activity. This further supports previous evidence that demyelination may be a secondary degenerative process associated with glaucoma disease progression. Not only do these results provide evidence for potential disease mechanisms, but this is also the first study to quantify optic nerve demyelination in glaucoma postmortem tissue.

## INTRODUCTION

Glaucoma is a type of optic neuropathy, primarily affecting retinal ganglion cells (RGCs) whose degeneration is responsible for one of the leading causes of blindness worldwide [1, 2]. Although the pathophysiology surrounding glaucoma is not fully known, increased intraocular pressure (IOP) is the predominant risk factor in both primary forms of glaucoma: open-angle and angle-closure glaucoma. Although caused for different reasons, the imbalance of aqueous humour dynamics within the eye and subsequent heightened IOP is suggested to strain and mechanical stress on axons. However, glaucoma related optic neuropathy can also be found in patients with normal IOP, known as normal tension glaucoma (NTG), and many people with elevated IOP do not develop glaucoma [1, 3].

The process of RGC degeneration in glaucoma is also not wholly understood, but some mechanisms have been identified as playing key roles in this process. Due to the multifaceted nature of glaucoma, it is difficult to identify a specific gene or biochemical mechanism, and instead multiple key factors work together to create the environment for degeneration. For example, RGC axonal dysfunction is a known hallmark of early progression in glaucoma, wherein anterograde and retrograde axonal transport is decreased [3, 4] Deficits or blockage of axonal transport can lead to environmental stressors on RGC’s such as excitotoxicity from increased glutamate, or deprivation of neurotrophic factors [4]. Another theory regarding the processes of degeneration in glaucoma involves demyelination, or the injury to the outer myelin sheath of neurons within the optic nerve, which may degrade the structural integrity and transmission efficiency of the neuron [2]. Though not fully understood, there are many possibilities as to how demyelination may occur in glaucoma. Oligodendrocytes (OLGs), which are responsible for myelinating axons, have been found to decrease in animal models of glaucoma[2, 5-7]. In an animal model of glaucoma, Nakazawa and colleagues [5] reported that upon inducing high IOP, there was a corresponding upregulation of tumour necrosis factor-α (TNF-α) as well as an increase in activated microglial expression. This may create a cytotoxic environment, as microglia expressing TNF-α specific receptors attack and injure OLGs and indirectly have a detrimental effect on RGCs. Although demyelination may not be the primary pathology observed in glaucoma, the neurons of the visual pathway are highly dependent on myelin integrity [8]. Due to the integral role myelin plays in the structure and upkeep of these neurons, demyelination can facilitate RGC death. This has also become a source of further research for therapeutics that have restorative or protective effects for myelin, such as targeting macrophage mediated inflammation or muscarinic receptors on OLGs [9, 10].

Despite multiple previous studies showing different aspects of how demyelination may promote RGC death in glaucoma, there is debate as to whether OLG and myelin injury may cause or is the effect of RGC damage. Many studies have found evidence that dysfunction to myelin structures and/or myelin related proteins occurs prior to RGC injury in glaucoma models and based on clinical evidence from glaucoma patients [5, 11, 12]. Contrariwise, the effects of demyelination have only been found in some studies until after RGC injury or death has occurred. This is the case for Son and colleagues [6], who similarly to Nakazawa and colleagues found astrocyte activation, activated microglia, and OLG loss in DBA/2J mice. This mouse strain was genetically selected and inbred to produce hereditary glaucoma and its corresponding symptoms similar to that in human glaucoma [13]. However, the increase in astrocyte reactivity was observed as an early response in the animal disease progression, and OLG loss did not occur until after the majority of axons and RGCs were already depleted. Comparable results have been found in other forms of animal glaucoma models including experimental autoimmune glaucoma (EAG). The effects of this model on the retina and optic nerves, including activation of autoimmune complement systems, occurred prior to any damage to myelin proteins (myelin basic protein, MBP) [14].

Most evidence of demyelination has been found indirectly through imaging studies in glaucoma patients, including the use of diffusion tensor imaging (DTI) [11, 15]. These studies reveal that there is some dysfunction or abnormalities to the optic nerve including increased diffusivity and decreased axonal integrity, indicating that demyelination has occurred. These measurements can even be correlated to the severity of glaucoma which further strengthens the argument for demyelination occurring in glaucoma [15]. Direct measurements of demyelination are difficult to ascertain in glaucoma patients, and the supply of appropriately collected postmortem tissue that is available for researchers to use is low [16]. The aim of this study was to be able to quantify the degree of demyelination in glaucoma using postmortem optic nerves from healthy and glaucoma patients. We used immunofluorescence to visualise MBP in the optic nerve as a marker for the presence or absence of demyelination. This was further correlated with optic nerve morphology, RGC survival, and the presence of reactive astrocytes (GFAP staining) and OLG transcription factors (Olig2 staining). This allows for a descriptive and elucidative idea of not only the extent of, but also the proposed mediators of demyelination in glaucoma.

## MATERIALS AND METHODS

### Postmortem Tissue

Following local ethics approval (reference no. 5201831944629) from Macquarie University Medicine and Health Sciences subcommittee, and in compliance with NHMRC HREA, postmortem eye samples were acquired from Lions NSW Eye Bank. These included the globe and attached optic nerve in preservation solution (formaldehyde, average 2 years and 4 months in fixative). Of these samples, the left and right eyes of four healthy (male n = 2, average age 71 years) and four primary open angle glaucoma (POAG) affected subjects (male n = 3, average age 80 years) were randomly selected for use (n=8 subjects, n=16 optic nerves). Details of the subjects and the tissue used are shown in the supplemental materials. The optic nerve, if not already removed, was cut from the most proximal area possible from the rest of the globe. It was then fixed in paraffin and cross-sections cut at 10μm thickness on a microtome (Mikrom HM325 Microtome).

Retinas from ten of the same corresponding eyes previously described (control n=5 and glaucoma n=5) were similarly prepared: first being cut from the rest of the globe such that the optic nerve head and peripheral retina were aligned. It was then cut on a microtome (above) at 7μm thickness. This use is further detailed in the supplementary materials.

### Immunofluorescence

Sections of optic nerve and retina underwent deparaffinising and rehydration in xylene and ethanol, respectively. This was followed by antigen retrieval in a citric acid buffer (pH 6) with Tween-20 (Sigma-Aldrich, 0.1% Tween 20 in PBS) at 60C for 45 minutes. Permeabilization was enhanced by applying 0.1% Triton X-100 (Lomb Scientific Pty Ltd, 0.2% Triton X-100 in PBS), and then blocked for 90 minutes to 2 hours at room temperature. The blocking solution included donkey serum in PBS and 0.3% Triton X-100. Sections were incubated with the selected primary antibodies, bovine serum albumin (BSA), and 0.3% Triton X-100 overnight at 4C. The antibodies utilised are listed in the following subsection (2.2.1). After being incubated with an appropriate secondary antibody and counterstained for DAPI for 1 hour, a mounting medium and coverslip was applied.

To ensure validity of the immunofluorescence results, certain controls were employed. This includes firstly choosing antibodies that have been validated either in previous studies or other experiments conducted within the same lab. Validating the antibodies for target specificity and minimising background staining was done by using control tissues of the same type and from different sources (animal). Further “practice” runs were performed after this to ensure the utilised antibodies were accurate in situ.

#### Antibodies

The primary antibodies used in this study are detailed as follows: MBP (1:500 Abcam - ab40390), Olig2 (1:1000, R&D Systems - AF2418), GFAP (1:1000, Dako - Z0334). The secondary antibodies were used with the appropriate primary and include donkey anti-rabbit Cy3 (1:500, Jackson ImmunoResearch - code 711-105-152) and donkey anti-goat Alexa 488 (1:500, Jackson ImmunoResearch - code 705-546-147), and counterstained using DAPI (1:1000, Invitrogen - ref. D1306).

The retinas were stained for NeuN (1:500, Abcam - ab104224), and only NeuN and DAPI double positive cells were considered for the purposes of RGC counting. The secondary antibody used in the retina was donkey anti-mouse Alexa 488 (1:500, Jackson ImmunoResearch, cat. 715-545-150) or donkey anti-mouse Alexa 647 (1:500, Jackson ImmunoResearch, cat. 715-605-151).

### Microscopy and Image Acquisition

Between four and six 10μm cross-sectional slices from eight optic nerves were stained and then imaged using the fluorescent microscope Zeiss Axio Imager 2. All imaging parameters, including light intensity (100%), binning (1, 1) and fluorescence exposure time were based on the “automatic” settings and were kept constant across all samples analysed. Exposure time for specific channels at all magnifications were determined by using multiple control images with the “auto” exposure function in Zeiss Axio Imager 2 followed by manual fine tuning to ensure the lowest possible exposure time that rendered the most visibly comprehensive image. Tiled images of the entire cross-section were taken at 5X magnification and then stitched using Zeiss ZEN Blue 3.5 (RRID: SCR_013672). For MBP and morphology analyses, two representative images were taken from each slice, whereas Olig2 and GFAP analyses had six representative images taken at 20X magnification.

Representative images used in figures were chosen based on the overall quality of the image to exclude images that were blurry, had any occlusions or fragments in the image, etc. Images that could not be suitably cropped or resized without altering the quality or detail of the image were excluded from use in figures. Lastly, images were chosen that best represented the results from each assessment and were not altered to achieve this (except for the digital recolouring performed for Figure 4).

#### Optic Nerve Morphology (adapted from Pelot et al., 2020)

Optic nerve morphology analyses were adapted from Pelot and colleagues [17]. The surface area of the optic nerve was acquired from the 5X tiled images, and the optic nerve including the surrounding sheath of pia mater and the central artery was measured in ImageJ/FIJI [18]. The number of visible fascicles from the same image was also manually counted in ImageJ/FIJI.

The 20X representative images stained for MBP were used to determine the average fascicle area: each image was converted to a greyscale image and individual fascicles were isolated and measured for surface area. The results from individual images were averaged ( $\frac{\mathrm{sum of products}}{\mathrm{number of products}}$). Due to the variability of sample quality, some subjects were excluded from certain analyses to avoid prejudicing the results. This includes one left eye and one right eye from two subjects within the glaucoma group. Therefore, the analyses for optic nerve surface area, optic nerve fascicle number, and optic nerve fascicle surface area, includes six glaucoma optic nerves (n=6) and eight control optic nerves (n=8).

The effective diameter of the optic nerve, fascicle, and connective tissue and septa were determined by equating a circle with the same surface area, as referenced in Pelot and colleagues (2020) and detailed below:

• Average Optic Nerve Area = ON
  Optic Nerve Effective Diameter = $2\times v(\frac{\mathrm{ON}}{p})$
• Overall Fascicle Surface Area (F) = average # of fascicle×average fascicle surface area
  Fascicle Effective Diameter = $2\times v(\frac{F}{p})$
• Septa Area (S) = *ON - F*
  Fascicle Effective Diameter = $2\times v(\frac{S}{p})$

The ratio of fascicle area and septa area to the overall optic nerve area was extrapolated by dividing their respective effective diameters. The proportionate change or difference between these two ratios was represented as a percentage and calculated as follows:

• ON:Fascicle ratio (FR) = F/ON
  ON:Septa ratio (SR) = S/ON
• Percent Comparison = $\left(\frac{\mathrm{FR}}{\mathrm{SR}}-1\right)\times 100$

#### GFAP/Astrocyte Morphology (adapted from Bosco et al., 2016)

Slides stained for GFAP were converted into a binary image in ImageJ/FIJI. The threshold level of each image was automatically derived using the equation:

$$\mathrm{threshold}=\frac{\left(\mathrm{average background}+\mathrm{average object}\right)}{2}$$

The pixels that fall above the average background threshold are made white in the binary image. This method of intensity-based thresholding and measurement was adapted from Bosco and colleagues [19]. This image was then skeletonised such that pixels along the edge of each object are removed until a single pixel “skeleton” of the object remains. The area of this skeleton is then measured for the GFAP positive area, as shown in Figure 5E.

#### Fluorescence Intensity

In Zen Blue 3.5, each image was split into individual channels (e.g. MBP, GFAP, Olig2), and converted to a TIF image type. This was then converted to an 8Bit image in ImageJ/FIJI, and the threshold of each image was computed as previously described. The mean intensity of each image is defined as:

$$\frac{\mathrm{sum of intensity}}{\mathrm{number of pixels}}$$

#### RGC Counting

Multiple images at 20x magnification were taken across the entire length of the slices of retina corresponding to five control and five glaucoma subjects whose optic nerves were also analysed. Only RGCs double stained for NeuN and DAPI was counted, which was then summed across each image of the entire retina to give a representative count of a subset of the retina. This was manually done in ImageJ/FIJI.

### Statistical Analyses

**Figure 1.**
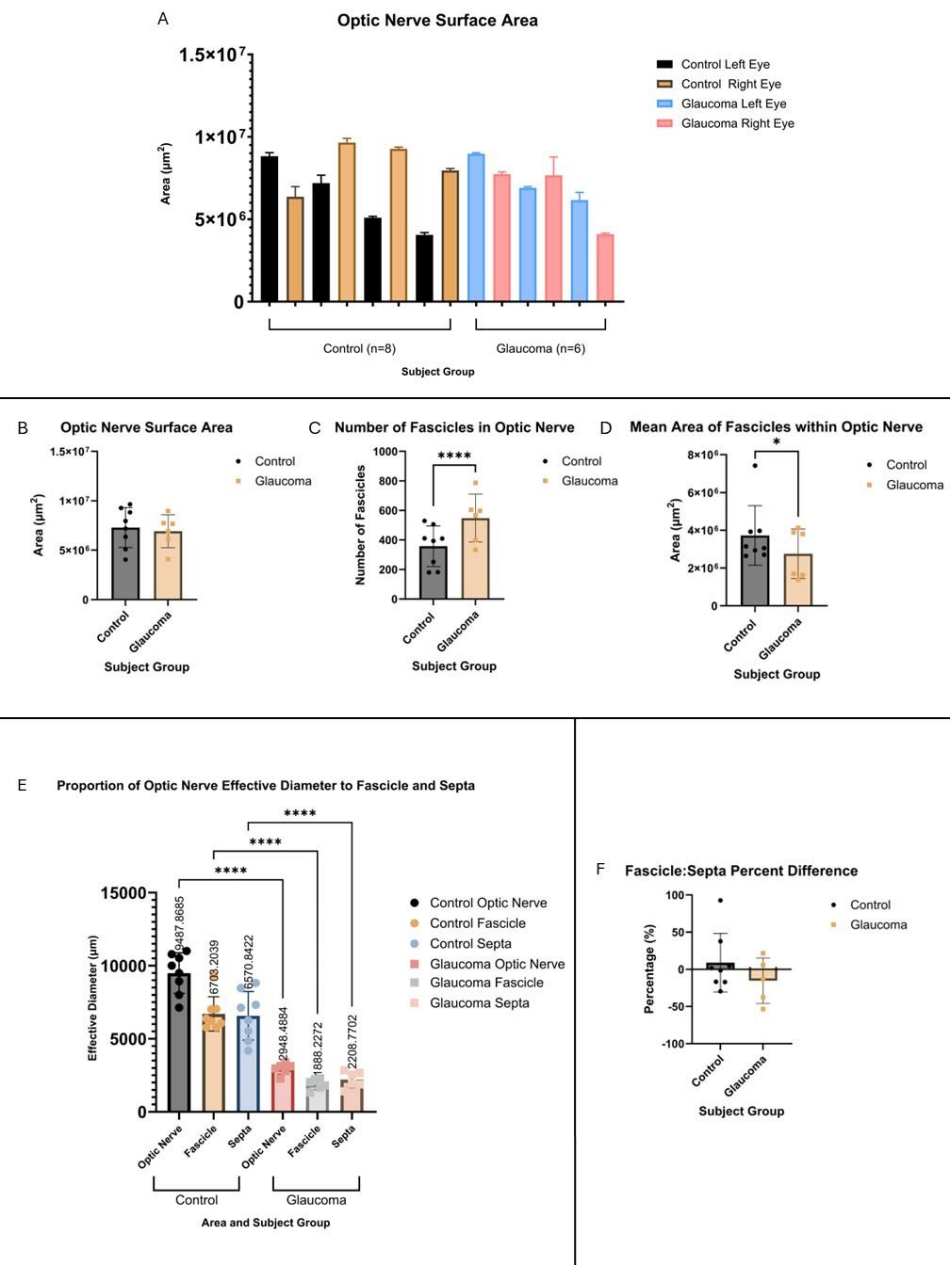
Morphological analyses of control and glaucoma subject eyes. (A) the optic nerve surface area of all analysed eyes (n = 16, replicates = 4-5) are presented here and labelled within the graph by their group (control, black and orange, or glaucoma, blue and pink) and the eye (left, black or blue, and right, orange or pink). This is done to show the variability of optic nerve size between groups and within subjects. (B) The optic nerve surface area between control and glaucoma subjects was not significantly different (unpaired t test). (C) The number of fascicle nerve bundles counted from each optic nerve was significantly higher in the glaucoma than the control subjects (unpaired t-test, p <0.0001). (D) The overall area of nerve fibres (average fascicle area x number of fascicles) was significantly smaller in glaucoma subjects when compared to control (Mann-Whitney exact p = 0.0018). (E) The proportion of optic nerve contents (nerve fibre fascicles or connective tissue and septa, coloured per legend within figure) was significantly greater for all control variables than the corresponding glaucoma (ordinary one-way ANOVA, all values p<0.0001). Furthermore, the percent comparison of the ratios of fascicle and septa area within the optic nerve revealed a greater, positive relationship for control optic nerves compared to glaucoma (unpaired t-test). (F) In all figures, the control group is represented in black with circular icons as applicable, and the glaucoma group is represented by orange, square icons, unless otherwise stated in the figure legend and/or caption. All p-values in figures are presented as * = p ≤ 0.05; ** = p ≤ 0.01; *** = p ≤ 0.001; **** = p > 0.0001. All values represented graphically are mean ± SD unless otherwise stated in the figure caption. Values for E is presented as mean diff ± SE.

Statistical analyses on two groups (i.e. control versus glaucoma) were performed using unpaired T-test in GraphPad version 10.1.2 [20], with results represented as mean ± standard deviation (SD), followed by the p-value. All other operations between multiple groups (two or more) were conducted as Ordinary One-Way ANOVA followed with Tukey’s multiple comparison test, as shown in figures 1D and 3B. This was also represented in text by the mean absolute difference (diff) ± standard error of difference (SE), and p-value. All statistical tests that render a significant result (p ≤ 0.0500) will have the corresponding 95% confidence interval listed after it in the format CI 95% lower, upper. Statistical tests for data sets that are not normally distributed (three instances) have been performed using Mann-Whitney tests, and are reported with the mean ± sd, Mann-Whitney p-value (exact or approximate), and confidence interval as appropriate. These instances have been identified within the text.

Within figures, certain types of data sets are represented differently. For example, to convey a set of replicates from one subject or analyses a bar graph showing mean and SD is utilised. All other figures representing multiple data sets from multiple subjects are in a scatter bar plot, with the data points from individual subjects plotted along with the mean and SD.

Correlations were visually represented by plotting the given variables in GraphPad. The generalised estimating equation (GEE) was then calculated in IBM SPSS Statistics version 28.0.0.0. This allowed consideration for within-subject variables as age and sex which would otherwise be overlooked. These results are given by their beta coefficient (B) and p-value.

## RESULTS

### Axonal degeneration in glaucoma manifests in optic nerve changes

**Figure 2.**
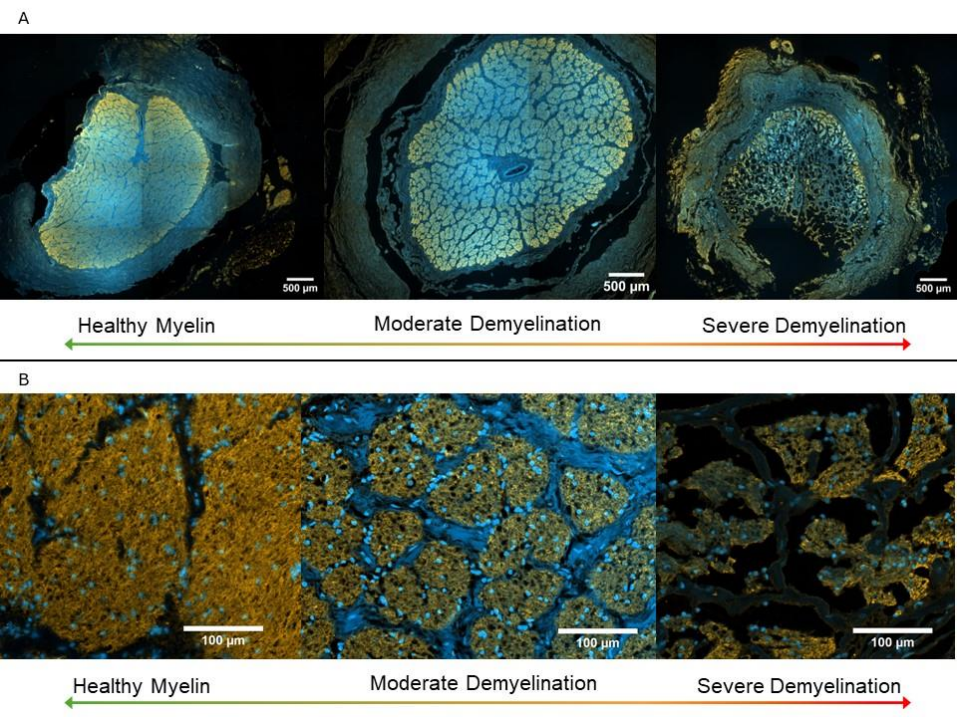
Representative immunofluorescent images from control and glaucoma optic nerves. (A) 5xmagnification tiled images of optic nerve cross sections stained for MBP (yellow) and DAPI (blue), with control to the left and more severely degenerated glaucoma to the right. The right image displays Schnabel’s cavernous degeneration which is characteristic of some more advanced cases of glaucoma. (B) 20x magnification representative images of nerve fibre fascicles displayed similarly to (A) such that control is to the left and more severely degenerated fascicles are to the right. Scale bars at the bottom right of each image indicate 500 μm (A) and 100 μm (B).

The area of the optic nerve in both glaucoma and healthy subjects revealed high variability between the left and right eyes, with some being significantly larger on one side than the other (Fig. 1A, Fig. 2A, n=16). As a whole, there was a slight but not significant increase in optic nerve surface area in healthy eyes when compared to glaucoma eyes (Mann-Whitney exact p = 0.1550) (Fig. 1B). This slight difference could be attributed to the variability between groups (control vs glaucoma) and within subjects (left eye vs right eye).

The fascicles or bundles of nerve fibres were counted from 5x magnification images of the entire optic nerve where possible and were then isolated from 20x magnification images of the optic nerve in ImageJ/FIJI to determine the area. There were significantly more fascicle bundles in the glaucomatous optic nerve (591.14 fascicles ± 61.64 vs 357.97 fascicles ± 48.51, p < 0.0001, CI 95% 94.71, 247.7) (Fig. 1C, Fig. 2B). However, the size of the fascicles were significantly smaller in surface area (2589438 μm^2^ ± 1463024 vs 3862228 μm^2^ ± 2534216, Mann-Whitney exact p = 0.0018, CI -1692844, -520433) (Fig. 1D, Fig. 2B).

Based on previous analyses on optic nerve morphology done by Pelot and colleagues (2020), the effective diameter of various components of the optic nerve was ascertained by fitting a circle with the same area as the surface area of the irregularly shaped optic nerve. This allows for a more direct comparison between certain parts of the optic nerve, including the fascicle area and septa/connective tissue area. The effective diameter of the optic nerve, fascicular area, and connective tissue areas were all significantly larger in control than glaucomatous patients (diff 9488μm ± 1386, 6703 μm ± 1176, and 6571 μm ± 1661 respectively) (Fig. 1E). The percent comparison of the ratios of fascicle to septa area within the optic nerve in control (8.985% ± 39.37) was revealed a greater, positive relationship in contrast to that of the glaucoma optic nerves (-15.32% ± 30.65) (Fig. 1F, Fig. 2A and B).

**Figure 3.**
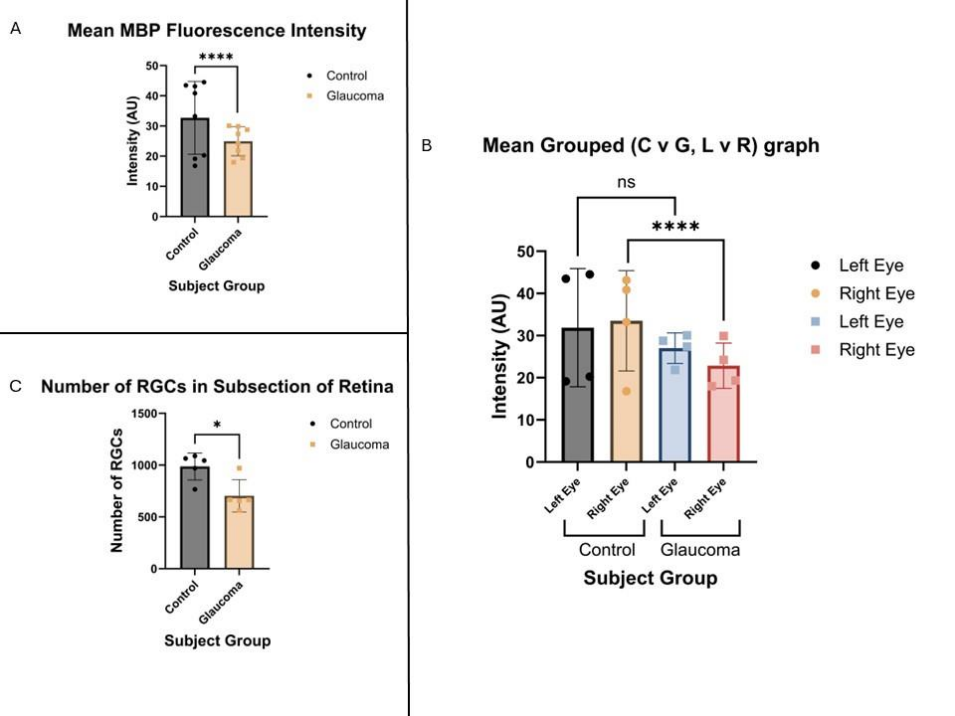
Myelin expression is significantly decreased in glaucoma subjects. (A) the mean fluorescence intensity for control (n = 8) versus glaucoma (n = 8) subjects is significantly higher (unpaired t-test, p<0.0001), and there were further differences when comparing left to right eyes between groups (coloured per legend within figure) (ordinary one-way ANOVA, right eye comparison p <0.0001, left eye comparison n.s.) (B). (C) The number of RGCs counted in subsections of the retina were significantly lower in glaucoma subjects (n = 5) than control (n = 5) (Mann-Whitney test, exact p = 0.0238). This was negatively correlated with the MBP fluorescence intensity (not shown). Values for B is presented as mean diff ± SE.

### Myelin basic protein levels are significantly diminished in glaucoma patients

The fluorescence intensity for MBP was measured in cross-sections of the optic nerve and was compared within groups and across subjects. There were some variations between fluorescence intensity between left and right eyes of the same subjects, however this was not significant. Taking the average fluorescence of a single pixel from each image, healthy optic nerves had higher immunoreactivity than glaucomatous optic nerves (32.30AU ± 14.55 vs 24.09AU ± 7.668, p < 0.0001, CI 95% -12.19, -4.229) (Fig. 3A). When comparing eyes, e.g. left control eye compared to left glaucoma eye, there were some differences between the two with the glaucomatous eye having less intensity (diff -4.622AU ± 2.603, p = 0.0800) (Fig. 3B). This was also found when comparing control right eyes to glaucomatous right eyes, which revealed a significant difference (diff -13.29AU ± 3.100, p < 0.0001, CI 95% -19.50, -7.078) (Fig. 3B).

To determine if there is a correlation between RGC loss in glaucoma and demyelination, the number of RGCs from five representative healthy and glaucomatous subjects were counted and plotted against the mean MBP intensity. There were significantly more RGCs counted in the subsections of control retina than glaucoma (986.4 RGCs ± 130.9 vs 703.4 RGCs ± 155.4, Mann Whitney exact p = 0.0238, CI -483.0, -74.00) (Fig. 3C). There was a slight, positive correlation between the number of RGCs and the mean MBP fluorescence (B = 0.001) which was not significant (p = 0.966) (not shown).

### Exploratory analyses of demyelination in glaucoma

**Figure 4.**
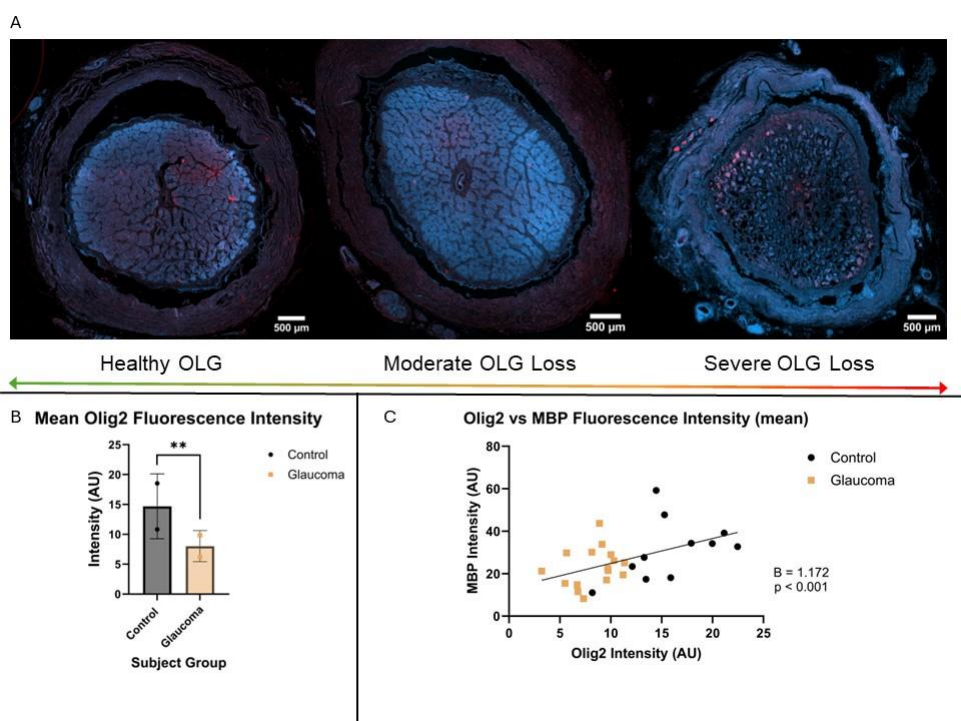
Oligodendrocyte precursors expression is significantly deteriorated in glaucomatous optic nerves. (A) Representative images of control and glaucoma optic nerves are shown to demonstrate the nature and appearance of Olig2 staining (digitally coloured red) in healthy and glaucomatous optic nerves. The control (left) image shows bright red patches of Olig2 staining within the optic nerve, in comparison to the glaucoma (middle and right) which has some sparse red staining. Scale bars at the bottom right of each image indicate 500um. (B) Control (n = 2) optic nerves had significantly higher fluorescence intensity for Olig2 than glaucoma (n = 2) (unpaired t-test, p = 0.0011). (C) When correlating Olig2 expression with MBP, there was a significant positive correlation between the two as shown by the plotted line of best fit and corresponding GEE beta coefficient (GEE, B = 1.172, p <0.001). The beta coefficient (B) and p-value for C are shown next to the graphs.

Two representative subjects from the control and glaucoma groups (n=4) were utilised for further immunofluorescence staining to determine if there is any correlation between potential methods of degeneration in glaucoma. Oligodendrocyte transcription factor (Olig2) had significantly higher fluorescence intensity in control than glaucoma representatives (14.14 AU ± 5.063 vs 8.432 AU ± 2.464, p = 0.0011, CI 95% -8.901, -2.507) (Fig. 4A and B). When plotted against the mean MBP fluorescence, there was a significant positive correlation between the two factors (B = 1.172, p < 0.001, CI 95% 0.728, 1.616) (Fig. 4C).

**Figure 5.**
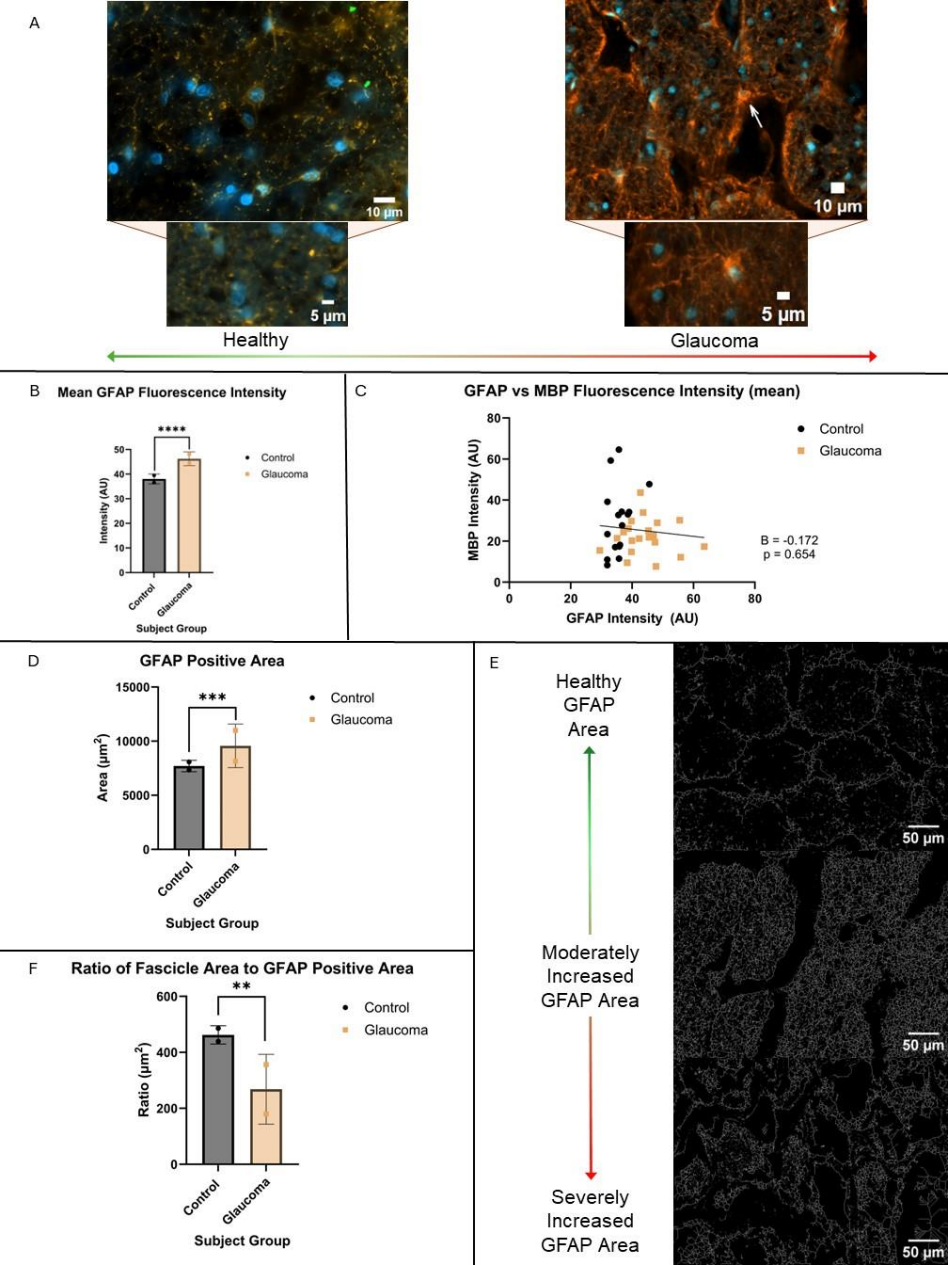
More active astrocytes and increased GFAP expression in glaucoma optic nerves. (A) representative images from healthy GFAP expression (left, yellow) and glaucoma (right, orange) taken at 40x magnification (top) with zoomed in images of individual astrocytes below. These images show the morphology of the multiple active astrocytes (right) in the glaucomatous optic nerve featuring extensive, thick branches and some atrophied astrocyte bodies (white arrow), in contrast to the quiescent astrocytes (left) with few, lightly stained processes in the control optic nerves. Scale bars at the bottom right of A indicate 10um (top) and 5um (bottom). (B) The fluorescent intensity for GFAP was significantly greater in glaucoma (n = 2) subjects than control (n = 2) (unpaired t-test, p <0.0001). (C) When plotting MBP fluorescence intensity against GFAP, there was a slight negative correlation per the GEE beta correlation, however this was not significant (GEE, B = -0.172, p = 0.654). (D) The nerve fibre area that was occupied with GFAP positive astrocyte processes were significantly greater in glaucoma than control subjects (unpaired t-test, p = 0.0005). This is shown in the skeletonised images (E) of the GFAP positive area with control optic nerves on top, contrasted by the dense mesh of GFAP positive astrocyte processes in the middle and bottom images. Scale bars at the bottom right of all images show 50 μm. Furthermore, the ratio of nerve fibre area to GFAP positive area (F) was significantly higher in healthy control optic nerves indicating there is more GFAP positively stained areas within nerve fascicles in glaucomatous optic nerves (unpaired t-test, p = 0.0011). The beta coefficient (B) and p-value for C are shown next to the graph.

Glaucoma optic nerve cross-sections from the same representative subjects as described above had significantly brighter GFAP fluorescence (48.64AU ± 7.406 vs 36.97 AU ± 5.397, p < 0.0001, CI 95% 9.048, 14.29) (Fig. 5A and B, Fig. 4A in red). This also resulted in a negative correlation when plotted against mean MBP (B = -0.172), however it was not statistically significant (p = 0.654) (Fig. 5C).

When observing GFAP staining for each subject, it became apparent that there were more reactive astrocytes in glaucoma subjects than in control when observing its morphology. This was quantified by taking the area of GFAP staining across the optic nerve, revealing that glaucomatous subjects had a greater area of active astrocytes than healthy subjects (9854 μm^2^ ± 1645 vs 7707 μm^2^ ± 684.7, p = 0.0005, CI 95% 1062, 3232) (Fig. 5D and E). Further, the ratio of overall fascicular area to GFAP positive area was significantly greater for control subjects (462.4 μm^2^ ± 73.66) than the same ratio for glaucoma subjects (268.0 μm^2^ ± 112.1, p = 0.0011, CI 95% -296.1, -92.67) (Fig. 5F).

### A case study in asymmetrical glaucoma

**Figure 6.**
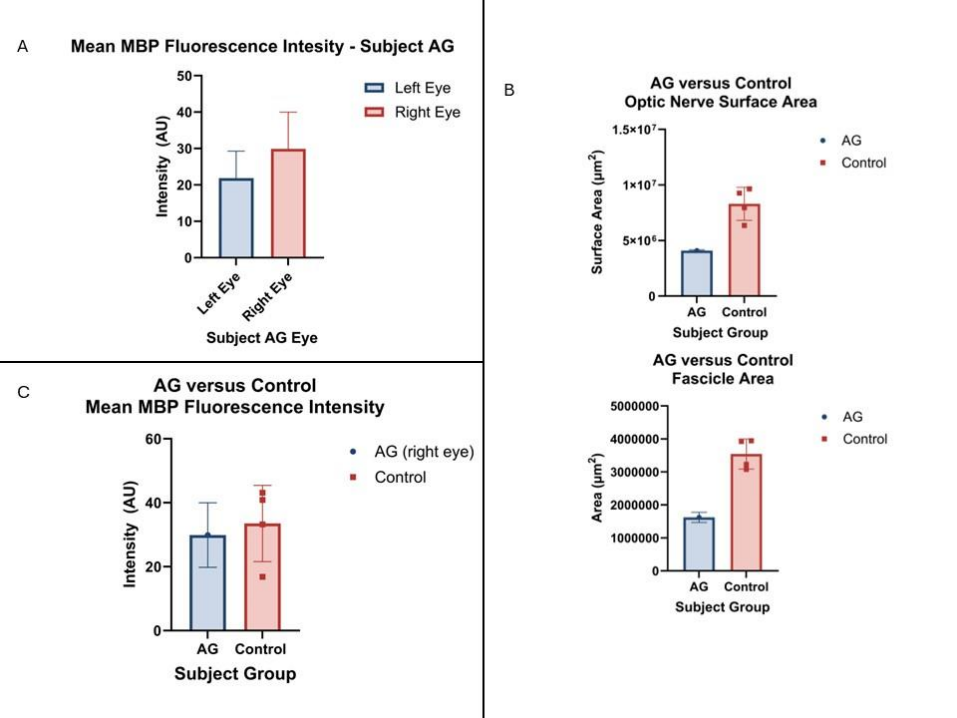
Asymmetrical glaucoma reveals decreased MBP expression with morphological changes. (A) the left glaucomatous eye (blue, replicates = 6) had less MBP expression than the right preperimetric eye (red, replicates = 5). However, the right eye was more similar in morphology to glaucomatous subjects than other control right eyes (n = 4). This includes decreased optic nerve surface area and nerve fibre/fascicle area (B, blue bars) when compared to controls (B, red bars). The preperimetric eye also exhibited MBP levels more similar to controls.

To determine if there are any effects to the contralateral eye in rare cases of asymmetrical glaucoma, the MBP fluorescence intensity was compared between the eyes of one subject with glaucoma presenting in the left eye and preperimetric glaucoma in the right eye. This is an example of asymmetrical glaucoma and was dubbed subject AG. It was found that the left glaucomatous eye had decreased MBP fluorescence intensity than the less severely affected eye (21.84 AU ± 7.426 vs 29.88 AU ± 10.11) (Fig. 6A).

It was also observed that certain aspects of the contralateral (preperimetric) eye were more similar to glaucoma subjects than healthy. For example, the overall optic nerve area and fascicular area were both decreased for AG’s right eye when compared to the right eyes of control subjects (Fig. 6B). This is consistent with observations that the optic nerve and fascicles are smaller in glaucoma than their healthy counterparts, as previously mentioned. However, there was no difference between the MBP fluorescence intensity when comparing the preperimetric right eye with control eyes (29.88 AU ± 10.11 vs 33.51 AU ± 11.91), further supporting that demyelination in glaucoma is related to the disease progress (Fig. 6C).

## DISCUSSION

The results of this study corroborate with many clinical assessments and animal model studies have suggested: that the optic nerve experiences demyelination in glaucoma and that this deterioration correlates with other cellular and biochemical deficits associated with glaucoma. Not only was this paired with morphological analyses of the optic nerve, but also with other indicators of modes of degeneration that may occur in glaucoma. Confirmation of demyelination in glaucoma

### Confirmation of demyelination in glaucoma

One of the main findings of this study was in confirming that myelin is significantly deteriorated in glaucomatous optic nerves than in healthy optic nerves. This study is also one of the first to quantify the degree of demyelination in postmortem tissue of glaucoma patients using immunofluorescent techniques. Although this has previously been elucidated to in normal, healthy optic nerves [21] and in other ocular conditions such as Leber hereditary optic neuropathy [22], very few if any histological assessments of demyelination have been conducted in postmortem glaucoma tissue. The immunofluorescent results from this research could be further confirmed by investigating changes in myelin proteins and other aggravating features of demyelination, such as Western blot analyses or enzyme-linked immunosorbent assays (ELISA).

These findings are supported by previous studies in animal models of glaucoma and by clinical observations in glaucoma patients that indicate demyelination as a potential key factor in glaucoma pathology [2, 11, 14]. For example, DTI measurements have been shown as a reliable measurement of axonal integrity loss and demyelination by indicating the diffusivity and direction of water molecules in the presence of axons [23]. In primary angle closure glaucoma (PACG), POAG, and normal tension glaucoma (NTG), it was found that they exhibited increased diffusivity and decreased anisotropy than normal [11, 15]. This evidence of axonal and myelin injury was also positively correlated with glaucoma severity measurements of Heidelberg Retina Tomography (HRT) based linear discriminant functions [15]. However, these measurements are not able to indicate the exact degree of demyelination, only the potential absence or presence of demyelination.

Animal studies have further suggested the role of demyelination in glaucoma models that has yet to be fully extended to human studies. Recently, Chaudhary and colleagues [24] confirmed that in nonhuman primates (rhesus macaques) with experimental glaucoma, the retrolaminar portion of the optic nerve has a significantly increased proportion of unmyelinated axons and smaller axons. Other animal models, such as novel combinations of mouse models of glaucoma (βB1-connective tissue growth factor + optic nerve antigen homogenate) allow for exploration of the effects of ocular hypertension and immune responses found in glaucoma [25]. This combination revealed less myelin staining (luxol fast blue, LFB) and decreased mRNA expression of myelin binding proteins. This finding was not replicated in either individual models of glaucoma, or in previous studies with the same optic nerve antigen homogenate model [14, 25]. This interesting finding implies that since only the glaucoma model which induces IOP elevation also induces demyelination, there is a dependent interplay between ocular pressure and myelin integrity. Although contradictory findings have been seen in some human analyses of NTG [2, 15] there are some explanations as to how ocular hypertension may influence RGC and myelin deterioration. Nogo-A, a myelin inhibitory protein, is significantly upregulated in the retinas of rats with ocular hypertension [7]. The regulatory role that Nogo-A performs for synapse maintenance and neuronal survival in normal retina is reversed when overexpressed as disruptions in synaptic function inhibit axonal regrowth and later can cause RGC death [7, 26].

The results of this study were able to find a slight but not significant correlation between RGC degeneration and demyelination when comparing the fluorescence intensity of MBP with the number of RGCs counted in a subsection of the retina. One main reason for this may be the small sample size available for comparison, as only ten eyes (five control and five glaucoma) were used for both analyses. Furthermore, more comprehensive analysis of axonal density could lend to more accurate numbers of surviving RGCs in glaucoma patients. Another reason for this is that in some cases the RGC body can be partially preserved or survive after axonal injury and demyelination occurs, and therefore the number of RGCs observed in the retina could be greater than the actual number of intact axons within the optic nerve depending on the course of disease [3]. Furthermore, the course of RGC atrophy can evolve over time following demyelination caused by inflammation and/or injury in optic neuritis cases, such that atrophy decreases with time, and it is possible that this is also the case in demyelination found in glaucoma [27].

### Morphological changes in glaucomatous retrobulbar optic nerve

Morphological changes to the optic nerve head via HRT measurements are more commonly analysed than the distal portions of the optic nerve in glaucoma patients [15, 28]. However, there are some studies that have analysed the diameter and cross-sectional area of the optic nerve. In POAG patients with and without ocular hypertension, echography scans showed that the diameter and cross sectional area in glaucoma patients was significantly smaller than that of healthy patients [29]. This was similarly confirmed in POAG patients presenting with various degrees of visual field defects, and also exhibited smaller optic nerve diameters than normal [30]. Interestingly, optic nerve shrinkage has been correlated with glaucoma severity as smaller optic nerve diameters are associated with worsening visual field defects and optic disc measurements such as the cup:disc ratio. This has been attributed to axonal degeneration and loss of optic nerve content found in glaucoma [28, 30]. Further studies could utilise these findings in further postmortem analyses coupled with patient clinical history to determine if there are any correlations between glaucoma severity, axonal loss, and demyelination.

Alongside overall optic nerve shrinkage, the fascicles or nerve bundles within the optic nerve also shrink as axonal degeneration advances. This results in a greater number of observed fascicles with smaller surface area, attributed to their disintegration into multiple, smaller bundles as observed here, as shown in Figure 2. This accounts for the change in ratio of nerve fibre area to overall optic nerve, and the ratio of septa and connective tissue within the optic nerve. This is especially true for the glaucomatous optic nerve exhibiting Schnabel cavernous optic atrophy. This morphological characteristic is denoted by dramatic loss of myelin and axons within the retrobulbar optic nerve (posterior to the lamina cribrosa) while the surrounding perimeter of septa remains, giving the appearance of empty caverns [31, 32]. This is a relatively rare phenomenon most commonly found in patients with glaucoma and/or uveal melanoma, but it can also be found in the optic nerves of patients with no reported ophthalmic disease or corresponding symptoms [31, 32].

### Relation between astrocyte reactivity and oligodendrocyte degeneration

Immunofluorescent analyses in this study included myelin proteins (MBP), reactive astrocytes (GFAP) and oligodendrocyte precursors (Olig2). These results corroborate previous literature regarding potential methods of degeneration and demyelination in glaucoma. For example, Olig2 fluorescence intensity was significantly diminished in glaucoma patients, and was positively correlated with MBP intensity. This identifies a potential relationship between decreased OLG precursors, a sign of demyelination, and the amount of myelin proteins. This has been previously observed in animal models of glaucoma, where the number of OLGs are significantly decreased and proliferation of OLG precursors (OPCs) is increased [5, 6]. Although this supports OLG degeneration having a main role in glaucoma demyelination, there is conflicting evidence regarding the timing and cause of this degeneration. There is also conflicting evidence as to whether astrocyte reactivity occurs before or after OLG degeneration, and therefore if astrocyte activation plays a causal role in demyelination. Supportive [5] and contradicting [6] results have been reported in studies utilising animal models of glaucoma. In an analysis of gene expression in a microbead induced ocular hypertensive model of glaucoma, Keuthan and colleagues [33] found that Olig2 gene expression was significantly decreased by three days post microbead injection. Although GFAP gene expression did not change significantly in the myelinated area of the optic nerve, it was significantly increased in the retinas of these animals. This is also supported by intraocular hypertension induced via anterior chamber perfusion, which saw significantly increased GFAP expression across the entire retina after 1 day [34]. Further studies would require more observation timepoints starting earlier to positively confirm the time course of OLG degeneration versus astrocyte activity, as observation times in previous studies range from one day to one week post injury or disease onset.

In this study, not only was GFAP fluorescence intensity significantly increased in glaucoma optic nerves, there was also a greater GFAP positive area indicating more active astrocytes. Although this is supported at surface level by previous studies (for example, [34], it does not necessarily support or disprove the possibility of changes to glial activity in demyelination. It is difficult to determine if only GFAP expression is beneficial or harmful, as the identification has expanded beyond “resting” and “active” stages [35]. This could be overcome in future studies by using more specific markers for known beneficial and harmful glial phenotypes [6, 25, 33].

### Evidence of transsynaptic degeneration in a case of asymmetrical glaucoma

Upon analysing a case of asymmetrical glaucoma, where the left eye was more severely affected, it was found that there was significantly diminished MBP fluorescence intensity than in the opposite eye. Furthermore, the right (preperimetric) eye had morphological features more similar to glaucomatous optic nerves rather than healthy, and also showed decreased MBP expression. This provides support that demyelination is one of many potential driving forces of neurodegeneration in glaucoma, which is associated with disease progression and severity. These results could be further expanded on in future studies with reliable postmortem samples from people with this type of asymmetrical glaucoma.

The spread of damage has been similarly reported in animal models of glaucoma. For example, after increasing interocular pressure in the right eye of rats, the optic tract of the contralateral (opposite) eye shrank [34]. Furthermore, damage was shown to spread transsynaptically into the higher visual centres of the brain. Evidence of apoptosis and astrocyte reactivity has been found in the dorsal lateral geniculate nucleus (dLGN) and superior colliculus (SC) in glaucoma and optic nerve injury animal models [12, 34]. Following this, there is evidence of increased neuronal atrophy and shrinkage of the dLGN in these animals and has also been seen in the LGN region of glaucoma patients [34, 36].

### Conclusion

The results of this study are one of the first to quantify the extent of demyelination in glaucoma patients, and further supports the association of demyelination in glaucomatous neurodegeneration. Future research could benefit from more longitudinal study structures, utilising clinical information regarding glaucoma severity and demyelination assessments, and further correlating this with postmortem analyses. This would overcome some of the limitations of this study, such as the small number of viable samples available for use. Another limitation is the limited access to patient medical history such as previous drugs and treatments, glaucoma disease severity, and relevant clinical information such as IOP. Further research could also support previous work regarding the potential pathological mechanisms underlying glaucoma, which are still not fully understood despite the wide prevalence of this disease. The implications of these findings can give light to new research areas and new potential therapeutic targets, such as remyelinating strategies.

## Supplementary Materials

The Supplementary data can be found online at: www.aginganddisease.org/EN/10.14336/AD.2024.0336.

## Data Availability

Manuscript open access and data repository availability will be made public when applicable.

## Software

ImageJ/FIJI; GraphPad Prism version 10.1.2 (GraphPad Software, Boston, Massachusetts, www.graphpad.com); Zeiss ZEN Blue 3.5 (RRID: SCR_013672); IBM SPSS Statistics (IBM)
