# Supplementary material for: Glaucoma, More than Meets the Eye: Patterns of Demyelination Revealed in Human Postmortem Glaucomatous Optic Nerve
Source: Aging Dis. 2024 Oct 1;15(5):2301–14. doi: 10.14336/AD.2024.0336-1 (PMC11346397; doi:10.14336/AD.2024.0336-1)
Supplement: Supplementary file 1 — The Supplementary data can be found online at: www.aginganddisease.org/EN/10.14336/AD.2024.0336. [file AD-15-5-2301-s.pdf]

SUPPLEMENTARY DATA

Glaucoma, More than Meets the Eye: Patterns of Demyelination Revealed in Human Postmortem Glaucomatous Optic Nerve

Gabriella E. Parrilla, Akanksha Salkar, Roshana Vander Wall, Vivek Gupta, Stuart L. Graham, Yuyi You

© 2024. Parrilla GE et al. Published online at http://www.aginganddisease.org/EN/10.14336/AD.2024.0336

SUPPLEMENTARY DATA

Table 1. Control Eye Samples – Subject Information.

Subject Number	Subject C1		Subject C2		Subject C3		Subject C4	
Age (years)	78		74		56		76	
Sex	Female		Male		Male		Female	
Time in Fixative (approx., in days)	~772 days		~774 days		~772 days		~772 days	
Time from death to time in fixative								
Experimental Use								
Subject Eyes	Left	Right	Left	Right	Left	Right	Left	Right
ON Morphology	X	X	X	X	X	X	X	X
RGC Analysis	X			X	X	X		X
Olig2 and GFAP Analysis		X				X		
Asymmetrical Glaucoma Comparison		X		X		X		X

Table 2. Glaucoma Eye Samples – Subject Information

Subject Number	Subject G1		Subject G2		Subject G3		Subject G4	
Age (years)	75		84		76		87	
Sex	Male		Female		Male		Male	
Time in Fixative (approx., in days)	~786 days		~1076 days		~876 days		~822 days	
Time from death to time in fixative								
Glaucoma Diagnosis	Primary Open Angle Glaucoma (POAG)							
Experimental Use								
Subject Eyes	Left	Right	Left	Right	Left	Right	Left	Right
ON Morphology	X	X	X			X	X	X
RGC Analysis	X			X		X	X	X
Olig2 and GFAP Analysis	X					X		
Asymmetrical Glaucoma Comparison							X	X

© 2024. Parrilla GE et al. Published online at http://www.aginganddisease.org/EN/10.14336/AD.2024.0336
